# Calib-RT: an open source python package for peptide retention time calibration in DIA mass spectrometry data

**DOI:** 10.1093/bioinformatics/btae417

**Published:** 2024-07-03

**Authors:** Yichi Zhang, Chenghui Hu, Xiaohui Wu, Jian Song

**Affiliations:** Pasteurien College, Suzhou Medical College of Soochow University, Soochow University, Suzhou 215000, China; Pasteurien College, Suzhou Medical College of Soochow University, Soochow University, Suzhou 215000, China; Pasteurien College, Suzhou Medical College of Soochow University, Soochow University, Suzhou 215000, China; Pasteurien College, Suzhou Medical College of Soochow University, Soochow University, Suzhou 215000, China

## Abstract

**Motivation:**

The data independent acquisition (DIA) mass spectrometry (MS) method is increasingly popular in the field of proteomics. But the loss of the correspondence between peptide ions and their spectra in DIA makes the identification challenging. One effective approach to reduce false positive identification is to calculate the deviation between the peptide’s estimated retention time (RT) and measured RT. During this process, scaling the spectral library RT into the estimated RT, known as the RT calibration, is a prerequisite for calculating the deviation. Currently, within the DIA algorithm ecosystem, there is a lack of engine-independent and readily usable RT calibration toolkits.

**Results:**

In this work, we introduce Calib-RT, a RT calibration method tailored to the characteristics of RT data. This method can achieve the nonlinear calibration across various data scales and tolerate a certain level of noise interference. Calib-RT is expected to enrich the open source DIA algorithm toolchain and assist in the development of DIA identification algorithms.

**Availability and implementation:**

Calib-RT is released as an open source software under the MIT license and can be installed from PyPi as a python module. The source code is available on GitHub at https://github.com/chenghui03/Calib_RT.

## 1 Introduction

The data independent acquisition (DIA) mass spectrometry (MS) method, which generates the MS/MS for all peptide ions within predefined windows, offers the advantages of high coverage and data completeness in peptide/protein identification (Gillet *et al.* 2012). While DIA avoids the randomness of peptide ion sampling in data dependent acquisition (DDA), it sacrifices the correspondence between peptide ions and their spectra, resulting in the possibility of peptide spectrum match (PSM) occurring multiple times over the whole liquid chromatographic (LC) gradient (Ludwig *et al.* 2018). This increases the risk of false positive identification. An evident strategy for identification optimization is to consider the retention time (RT) of target peptides. Only PSM, of which the measured RT is within a certain tolerance of the estimated RT, is considered a reliable identification. Generally, the identification engine can only access the RT of the peptide provided by the spectral library (including sample-specific library derived from DDA data or public library). Because the chromatographic systems used for library creation may be heterogeneous to a particular experiment, spectral library RTs cannot be directly used as the estimated RTs. Escher *et al.* (2012) proposed a method for RT normalization that transforms the RT of a peptide into an empirically derived dimensionless value, thereby facilitating its transfer across laboratories and chromatographic systems. Bruderer *et al.* (2016) expanded the anchor peptide set used for iRT transformation, demonstrating that the accuracy of iRT can improve the DIA identification. The iRT concept is also adopted by in silico spectral libraries which make use of deep learning predictors (Gessulat *et al.* 2019). When the spectral library provides iRTs for target peptides, it obviously cannot be employed to estimate RTs without further adjustment. As a result, the DIA identification engine has to perform RT calibration between the spectral library scale and the specific chromatographic system scale to estimate the RTs of any target peptides, which is referred as the RT calibration problem. Given the hard truncation of RT deviation used in the DIA identification process and the relative importance of the RT deviation score among dozens of subscores in DIA (in contrast, RT deviation score has minimal effect on DDA identification, Gessulat *et al.* 2019), executing the accurate RT calibration significantly influences the identification of DIA data (Bruderer *et al.* 2016).

Different DIA identification engines use different RT calibration methods. OpenSWATH (Röst *et al.* 2014) employs linear or polynomial regression based on the identified iRT peptides (Escher *et al.* 2012). Spectronaut (Bruderer *et al.* 2016) first introduced iterative analysis approach where RT calibration is enhanced based on current round of identification using a segmented local regression on an expanded set of iRT peptides. The iterative approach or the bootstrap strategy of Spectronaut was subsequently incorporated into MaxDIA (Sinitcyn *et al.* 2021) and DIA-NN (Demichev *et al.* 2020) in their own respective flavors. DIA-NN employs isotonic regression and spline curve fitting, while MaxDIA utilizes a nonlinear function (not disclosed). EncyclopeDIA (Searle *et al.* 2018) applies a nonparametric kernel density estimation calibration algorithm on the first round identified peptides with the help of a chromatogram library. MSFragger-DIA (Yu *et al.* 2023) follows a spectrum-centric approach to identify the most likely PSM for each mixed spectrum. It then performs RT fitting based on these PSMs using the local weighted regression (LOESS) and monotonic regression functions. Although these engines use different calibration functions on their respective selected data to achieve the RT calibration, these calibration methods cannot be used independently by the development community. This limitation hinders systematic research on the RT calibration problem and the evaluation of various calibration methods. Considering the significant impact of RT calibration on DIA identification, it is necessary to develop an engine-independent RT calibration algorithm. This algorithm should meet the requirement of the monotonic increasing constraint while being capable of removing noise from the fitted data, as both iterative and noniterative identification processes inevitably involve noise in the calibration data. The iteration-based identification methods (e.g. Spectronaut and DIA-NN) detect peptides on a subset of target peptides and update the RT calibration using the peptides at 1% false discovery rate (FDR). However, the FDR estimation of a subset of target peptides may underestimate due to sampling bias, resulting in actual noise exceeding the 1% FDR. For noniteration based identification methods (e.g. EncyclopeDIA and MSFragger-DIA), they do not estimate FDR but instead select peptides based on customed conditions or directly utilize all peptides from the spectral library as the calibration input, highly likely resulting in over 1% FDR levels of noise for calibration. Rosenberger *et al.* (2017) mentioned that the fraction of false target peptides in the spectral library may reach 0.75, indicating that the noise level is approximately at 75% FDR level. Therefore, it is crucial for an independent universal calibration algorithm to possess the capability for noise removal and fitting while adhering to the monotonic increasing constraint, even at noise levels up to 75% FDR.

In this work, we propose Calib-RT, a RT calibration method tailored to the characteristics of the calibration data. Our experiments demonstrate that this method is applicable to different data size and a certain levels of noise interference, achieving accurate transformation of retention time scale. We believe Calib-RT will enrich the open source DIA algorithm toolchain and assist in the development of DIA identification algorithms.

## 2 Workflow and datasets

### 2. 1 Workflow

**Figure 1.**
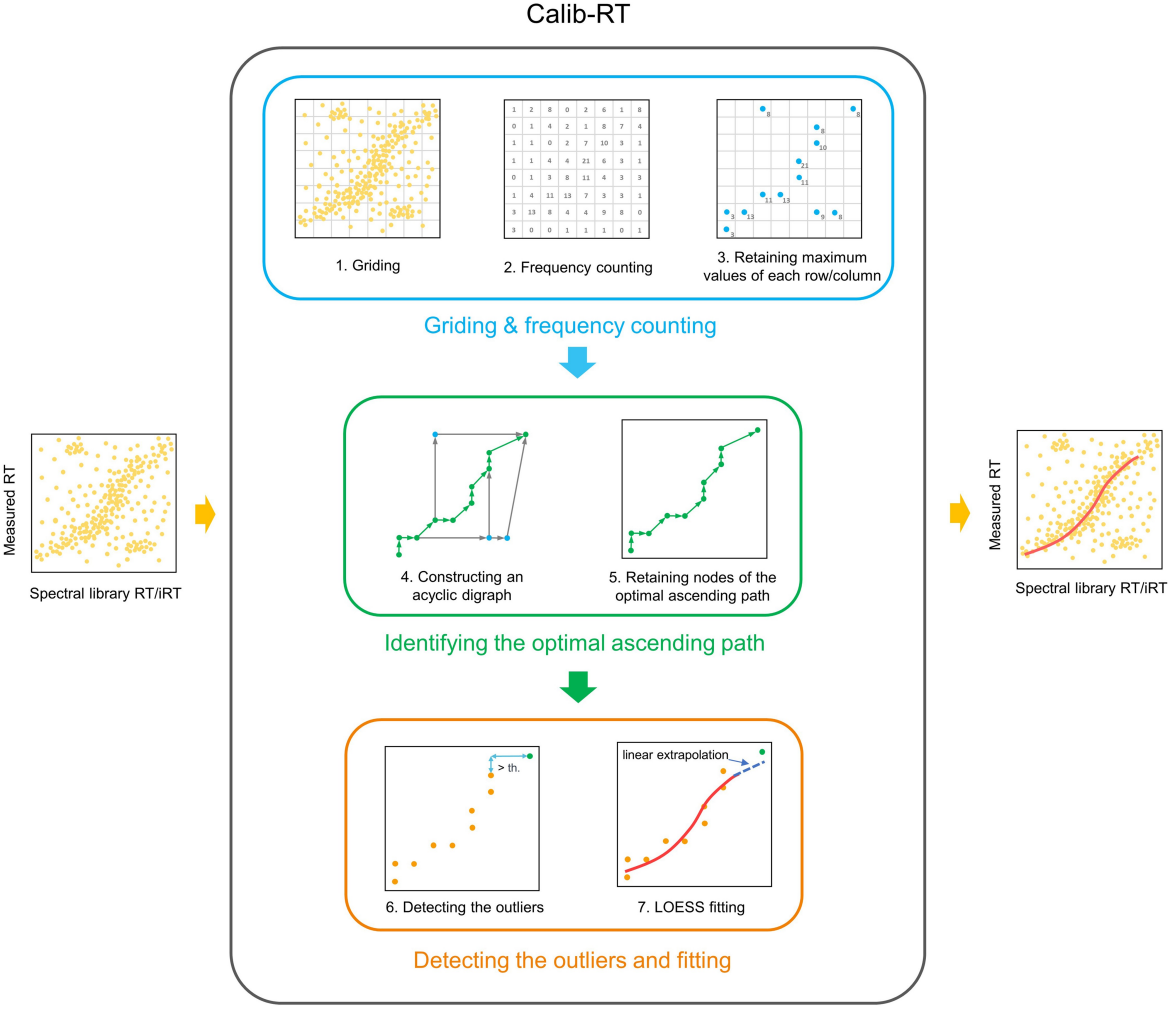
The workflow of Calib-RT.

The purpose of Calib-RT is to fit the peptides’ spectral library RTs/iRTs with their corresponding measured RTs, enabling the conversion of any spectral library RT/iRT to an estimated RT. On the one hand, it is possible for the peptides used in calibration to contain false positives. On the other hand, the relationship between the spectral library RT/iRT and measured RT exhibits a monotonically increasing pattern (Demichev *et al.* 2020). As a result, the RT calibration problem fundamentally pertains to a 1D nonlinear fitting challenge with the presence of noisy interference and the imposition of monotonic constraints. Taking these characteristics into account, Calib-RT designs the workflow (Fig. 1) below to carry out the calibration:

1. Griding and frequency counting of data points. For the sake of convenience in description, we refer to the spectral library RT/iRT and measured RT of a peptide as a data point on an library-measured-RT plane. Calib-RT grids the data points (by default 100×100) and counts the number of data points that fall into each grid cell. Then, Calib-RT retains the cell with the maximum value in each row or column. Griding and nonmaximal suppression effectively reduce the data size.
2. Identifying the optimal ascending path. Calib-RT creates a directed acyclic graph with weights, where the nodes are all the cells obtained in the first step. The edges of the graph must satisfy an increasing condition (meaning the terminal node must not be smaller than the starting node in both RT dimensions). The weight of the edge is the product of the node frequencies divided by the edge length. Calib-RT identifies the nodes corresponding to the path of maximum weight sum in the directed acyclic graph for subsequent fitting. Through this step, Calib-RT further filters out noise while satisfying the monotonicity constraint.
3. Detecting the outliers and fitting. Because the ground truth points are densely distributed in the center and sparsely at both ends, the points found in the previous step may reveal large spans at the ends. These wide-span points are more likely to be outliers. By setting an allowable span (10% of the whole range of both dimensions typically), potential outliers are filtered. Finally, Calib-RT uses LOESS to complete the calibration based on the filtered points and performs the linear extrapolation at both ends of the calibration curve. Of note, outliers detecting (or span filtering) is disabled by default.

### 2.2 Datasets

We empirically categorize the RT calibration curves into the following five types: linear, distortion at the beginning of elution, distortion at the end of elution, exponential and S type. Each type undergoes testing with two DIA-MS files which are retrieved from either the ProteomeXchange repository or in-house data (Supplementary Table S1). For each DIA file, DIA-NN’s library-free approach (v1.8.1) was used to report the identified precursors. The spectral library iRTs (predicted by DIA-NN) and measured RTs of the identified precursors at 1% FDR are considered as the noise-free ground truth values.

In order to comprehensively evaluate the calibration performance of Calib-RT under different data scales and various levels of noise interference, we sampled the ground truth points of each test dataset at different rates (i.e. sampling rate, SR, including 0.01, 0.1, 0.3, and 0.5). As the RT calibration should be performed before the final identification, the sample rates did not include 1. The sampled data were then combined with randomly distributed noise data, where the range of the noise data does not exceed that of the sampled data, resulting in noise levels at 1%, 5%, 35%, and 75% FDR (a noise level at x% FDR indicates that x% of the data points are noise out of the total number of data points). As a result, each original dataset was augmented into 16 datasets by applying sampling and introducing noise.

In addition, we used the mean relative deviation (MRD) between predicted RTs and measure RTs to evaluate the fitting performance which only considers the data points without noise.

## 3 Results

First, we checked the fitting results of Calib-RT using different grid sizes (25, 50, 100, and 200) on datasets with the most data points (Distortion-End-II, SR: 0.5, FDR: 75%, #points: 244 724) and the fewest data points (Exp-II, SR: 0.01, FDR: 1%, #points: 21), as shown in Supplementary Fig. S1. It can be observed that Calib-RT using the grid size 25 or 50 does not achieve the optimal MRD but 100 balances the MRD and computational complexity well, thus defaulting to a grid size of 100 for Calib-RT is reasonable.

The essence of Calib-RT lies in removing noise before conducting LOESS fitting. To evaluate its performance, we conducted a comparative assessment on test datasets of Calib-RT against three other methods: (i) Raw LOESS without noise removal (Raw-LOESS); (ii) Noise removal based on the error quantiles followed by LOESS fitting (Quantile-LOESS, Rodríguez *et al.* 2019); (iii) Noise removal based on the random sample consensus algorithm followed by LOESS fitting (RANSAC-LOESS). The principles, parameters and implementations of the three methods are detailed in Supplementary Note S1. The performance results are presented in Supplementary Fig. S2. It can be observed that when the noise level is at 1% FDR, all four methods can fit the data well. When the noise level increases to 5% FDR, Raw-LOESS and RANSAC-LOESS show fitting variations on Exp-II (SR: 0.01, FDR: 5%) and S-I (SR: 0.1, FDR: 5%) datasets, respectively. When the noise increases to 35% FDR, Quantile-LOESS exhibits fitting fluctuations on Distortion-End-I (SR: 0.1, FDR: 35%) and Exp-II (SR: 0.01, FDR: 35%). When the noise level reaches 75% FDR, Raw-LOESS, RANSAC-LOESS and Quantile-LOESS all fail to calibration. By contrast, Calib-RT is able to fit with minimal deviation at noise levels ranging from 1% to 75% across all datasets. Meanwhile, in datasets Distortion-End-I (SR: 0.01, FDR: 75%) and S-II (SR: 0.01, FDR: 75), due to the local noise density exceeding the signal density, there is a trivial variations by Calib-RT (with MRDs of 0.03 and 0.05, respectively). Besides, Calib-RT's execution speed is nearly unaffected by the data size (for all test datasets, its runtime is <0.1 s on an ordinary laptop), whereas RANSAC-LOESS, as a comparison, takes much longer, with a runtime of up to 128.90 s on the S-I (SR: 0.5, FDR: 0.75) dataset.

The noise data in the above experiments all fall within the range of true data points. Hence, Calib-RT disables the span filtering to equally consider all data points. However, if the noise range exceeds the range of true values, the fitting curve needs to balance the “inertia” of the fitting curve and the reliability of the “jumping” data points at both ends of the curve. As shown in Supplementary Fig. S3, where the noise iRT limit is 1.2 times the true data iRT limit, the span filtering can be activated (with 10% span threshold) to obtain a more reasonable result at both ends of the calibration curve. Span filtering indicates that the likelihood of linear extrapolation is greater than the likelihood of turning for both ends of the fitting curve.

In summary, Calib-RT outperforms generic fitting algorithms with noise removal across datasets of various sizes and noise interference, since it is tailored to the characteristics of the RT data.

## 4 Conclusion

We presented Calib-RT, an open source python software package for retention time calibration. Calib-RT achieves the goal of calibration across various data scales and a certain level of noise interference. We believe that our engine-independent Calib-RT will enrich the open source DIA algorithm toolchain and assist in the development of DIA identification algorithms.

## Supplementary Material

btae417_Supplementary_Data
