# Supplementary material for: Calib-RT: an open source python package for peptide retention time calibration in DIA mass spectrometry data
Source: Bioinformatics. 2024 Jul 3;40(7):btae417. doi: 10.1093/bioinformatics/btae417 (PMC11223842; doi:10.1093/bioinformatics/btae417)
Supplement: btae417_Supplementary_Data [file btae417_supplementary_data.pdf]

Supplementary information

Table 1. MS datasets used for the analysis.

ID	PRIDE ID	File Name	#Precursors (1% FDR)
Linear-I	PXD029836	20211101_PRO2_LS_04_MA_HeLaSCS_0.2_ngHS_GE2_1_1408	4510
Linear-II	PXD040205	20220714_10ngK562_ZI_500ul60C3cm5min_P1-C1_1_9675	3666
Distortion-Begin-I	PXD022216	fmeierab_T190525_CLL_diaPASEF_04_1979	53454
Distortion-Begin-II	PXD022216	fmeierab_T190525_CLL_diaPASEF_40_1965	47541
Distortion-End-I	PXD017703	20200505_Evosep_100SPD_SG06-16_MLHeLa_100ng_py8_S2-C1_1_2731	46053
Distortion-End-II	PXD029836	20211103_PRO2_LS_01_MA_HeLa_200_SDC_NS_RE2_1_1418	122358
Exp-I	In-house	In-house	3109
Exp-II	In-house	In-house	1844
S-I	PXD017703	20200505_Evosep_200SPD_SG06-16_MLHeLa_200ng_py8_S3-A1_1_2737	29491
S-II	PXD038828	CMs_Subject3_Lvmid_G10_BG11_1_7560	8892

Note 1: The principles, parameters and implementations of other three fitting methods.

Raw-LOESS: This method does not consider noise removal and directly fits using all data points. The key parameter, spanvalue, for LOESS is determined through five-fold cross-validation among six candidate values [0.05, 0.1, 0.15, 0.2, 0.25, 0.3]. LOESS and cross-validation methods are implemented using statsmodels (version: 0.13.5) and Scikit-learn (version: 1.2.2) respectively.

Quantile-LOESS: This method first optimizes the spanvalue parameter of LOESS based on the "an" information criterion (AIC, Akaike, 1974), then calculates the prediction error for all points. Next, it calculates the quantiles of the errors, and uses the Tukey criterion (Tukey, 1977) to identify outlier points whose values below Q1−1.5×(Q3−Q1) or above Q3+1.5×(Q3−Q1), where Q1 and Q3 represent the first and third quartiles respectively. After filtering out the outlier points, it performs the LOESS fitting again. This method is proposed and implemented by Rodríguez et al., 2019.

RANSAC-LOESS: This method is based on the Random Sample Consensus (RANSAC) approach to eliminate noise interference. Specifically, the algorithm utilizes Quantile-LOESS fitting on 20% of randomly selected data points, identifying inliers and outliers based on the median of fitting residuals. This process is repeated for up to 100 iterations, and the model with the maximum number of inliers is chosen as the final fitting model. The RANSAC algorithm is implemented using Scikit-learn (version: 1.2.2), and the implementation of Quantile-LOESS is the same as above.

References

1. Akaike H. A new look at the statistical model identification[J]. IEEE transactions on automatic control, 1974, 19(6): 716-723.
2. Tukey J W. Exploratory data analysis[M]. Reading, MA: Addison-wesley, 1977.
3. Rodríguez Ó, Pignata G, Hamuy M, et al. Type II supernovae as distance indicators at near-IR wavelengths[J]. Monthly Notices of the Royal Astronomical Society, 2019, 483(4): 5459-5479.

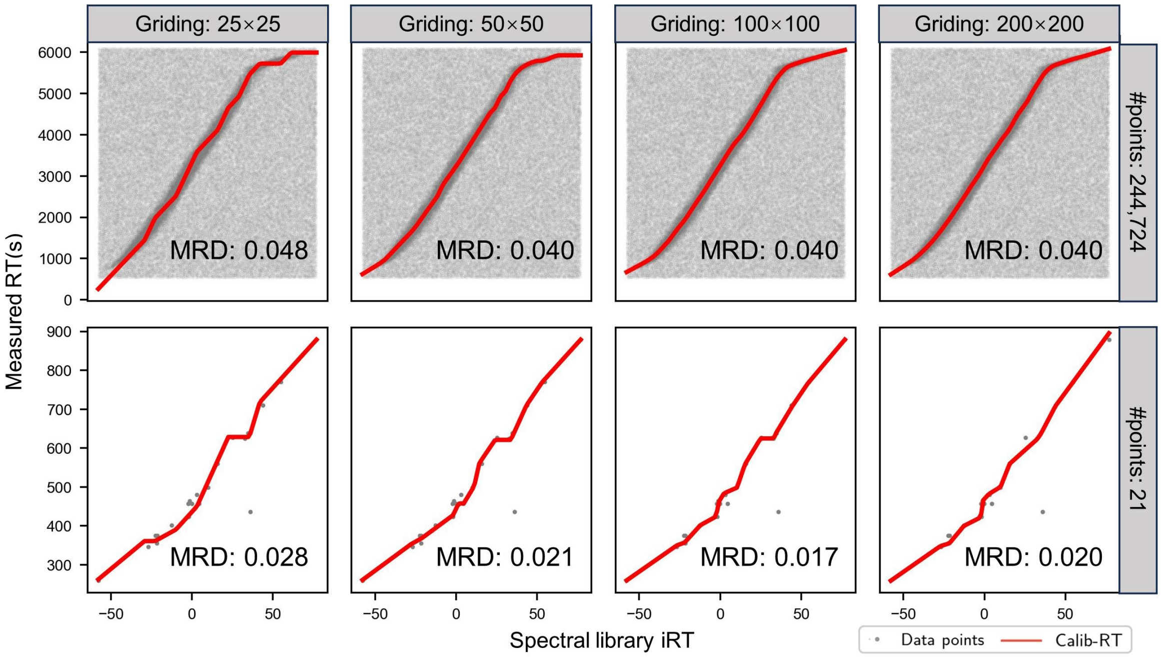

Fig. 1. The fitting results of Calib-RT using different grid sizes on datasets with the most data points (244,724, the first row subplots) and the fewest data points (21, the second row subplots).

a) Distortion-Begin-I dataset

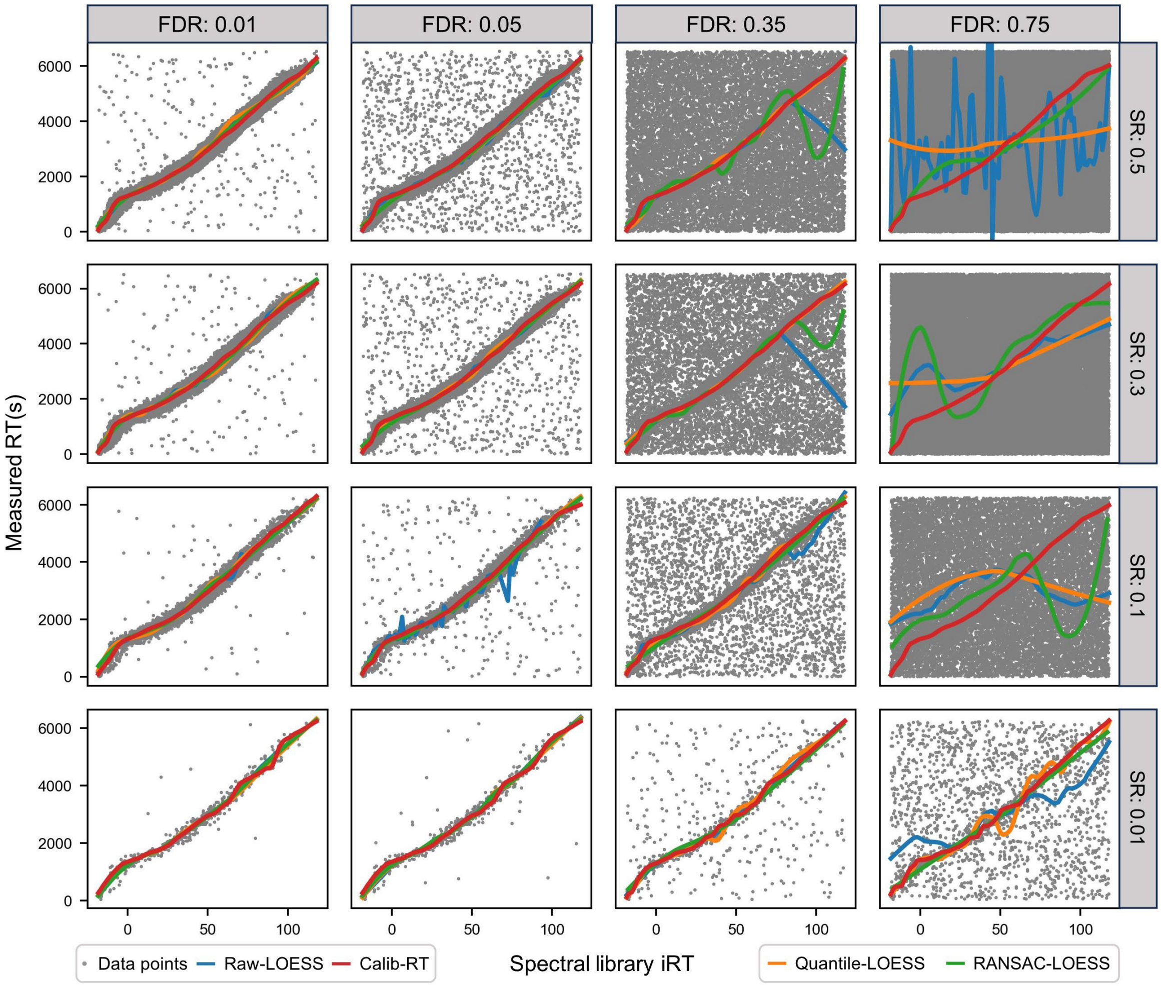

SR	FDR	#points	MRD				Time(s)			
			Raw-LOESS	Quantile-LOESS	RANSAC-LOESS	Calib-RT	Raw-LOESS	Quantile-LOESS	RANSAC-LOESS	Calib-RT
0.50	0.01	26,951	0.14	0.15	0.16	0.11	0.28	2.92	2.30	0.03
	0.05	28,086	0.13	0.16	0.15	0.11	0.28	2.59	9.87	0.04
	0.35	41,048	0.15	0.12	0.21	0.11	0.31	4.80	78.72	0.04
	0.75	106,724	1.96	2.07	0.26	0.11	0.28	1.44	77.58	0.05
0.30	0.01	16,171	0.14	0.14	0.15	0.14	0.23	3.24	3.72	0.03
	0.05	16,852	0.18	0.19	0.24	0.14	0.22	3.06	7.25	0.04
	0.35	24,630	0.36	0.30	0.24	0.14	0.23	4.65	78.35	0.04
	0.75	64,036	1.33	1.98	0.65	0.13	0.24	1.83	78.11	0.04
0.10	0.01	5,391	0.15	0.17	0.21	0.11	0.14	2.17	5.38	0.04
	0.05	5,618	0.15	0.12	0.12	0.10	0.14	2.34	7.62	0.04
	0.35	8,211	0.14	0.13	0.20	0.10	0.15	4.44	78.45	0.03
	0.75	21,348	1.26	1.38	0.74	0.10	0.14	1.37	79.58	0.03
0.01	0.01	541	0.08	0.08	0.07	0.09	0.11	2.65	2.16	0.03
	0.05	564	0.07	0.08	0.08	0.09	0.10	2.00	5.02	0.03
	0.35	824	0.08	0.08	0.11	0.07	0.10	4.72	78.54	0.03
	0.75	2,140	0.49	0.13	0.12	0.09	0.10	4.90	78.79	0.02

b) Distortion-Begin-II dataset

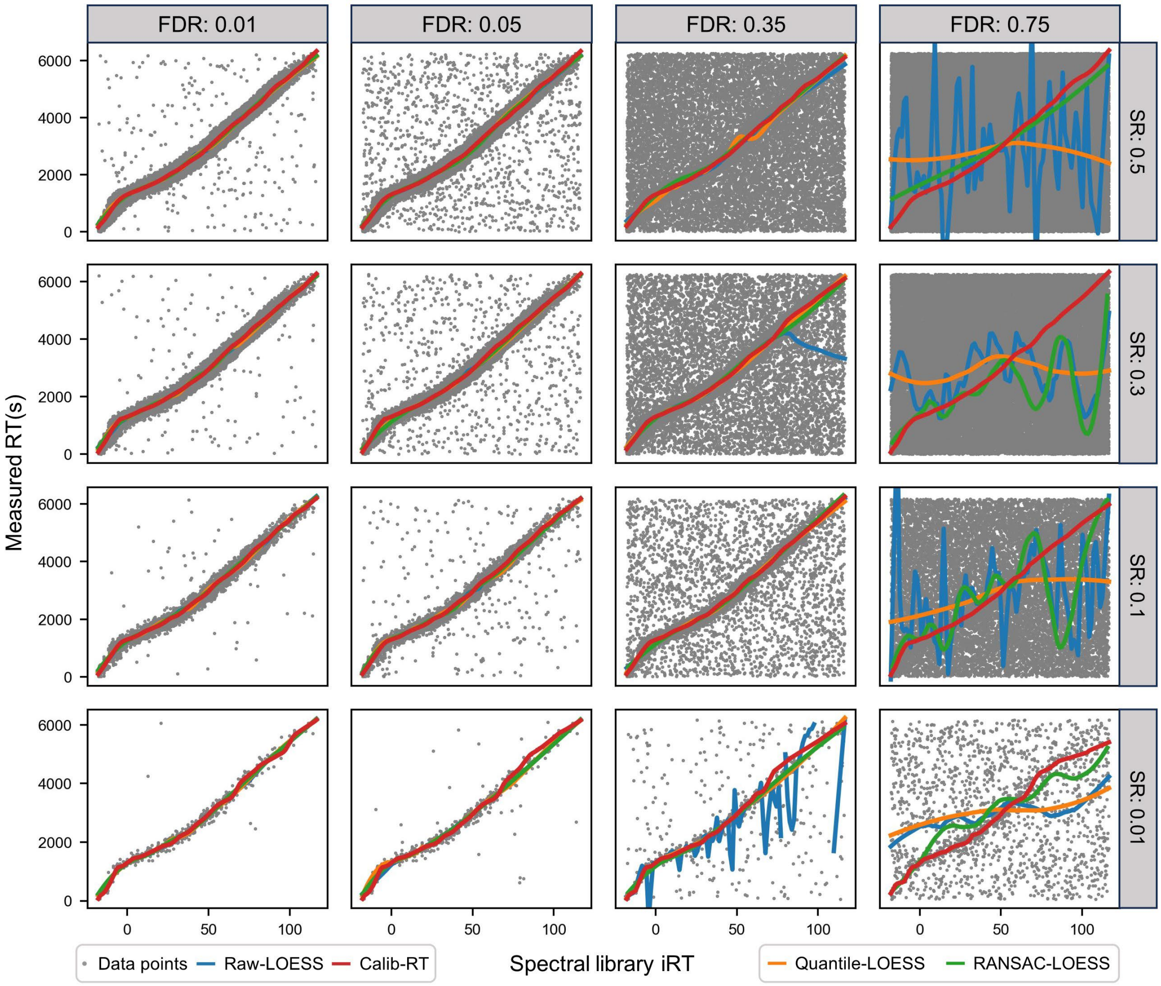

SR	FDR	#points	MRD				Time(s)			
			Raw-LOESS	Quantile-LOESS	RANSAC-LOESS	Calib-RT	Raw-LOESS	Quantile-LOESS	RANSAC-LOESS	Calib-RT
0.50	0.01	23,962	0.13	0.13	0.14	0.11	0.26	1.92	2.15	0.04
	0.05	24,971	0.12	0.12	0.12	0.11	0.28	2.22	5.18	0.04
	0.35	36,496	0.16	0.14	0.13	0.11	0.29	4.60	77.60	0.04
	0.75	94,888	0.98	1.15	0.48	0.11	0.28	1.42	78.55	0.04
0.30	0.01	14,378	0.12	0.12	0.12	0.09	0.22	2.18	3.63	0.04
	0.05	14,984	0.11	0.11	0.12	0.09	0.22	2.63	5.97	0.04
	0.35	21,899	0.13	0.13	0.12	0.11	0.22	3.62	77.94	0.04
	0.75	56,936	1.14	1.24	0.27	0.09	0.21	1.35	77.30	0.04
0.10	0.01	4,794	0.09	0.09	0.10	0.08	0.14	3.28	2.29	0.03
	0.05	4,996	0.08	0.09	0.09	0.08	0.15	3.20	5.96	0.03
	0.35	7,302	0.11	0.09	0.11	0.08	0.14	3.63	78.17	0.03
	0.75	18,984	0.98	0.70	0.29	0.08	0.15	1.40	78.73	0.03
0.01	0.01	481	0.07	0.07	0.07	0.06	0.10	4.00	3.47	0.03
	0.05	502	0.08	0.08	0.07	0.06	0.10	2.19	6.05	0.03
	0.35	733	0.15	0.06	0.08	0.06	0.10	4.05	78.07	0.02
	0.75	1,904	0.66	0.76	0.22	0.07	0.10	1.31	78.06	0.02

c) Distortion-End-I dataset

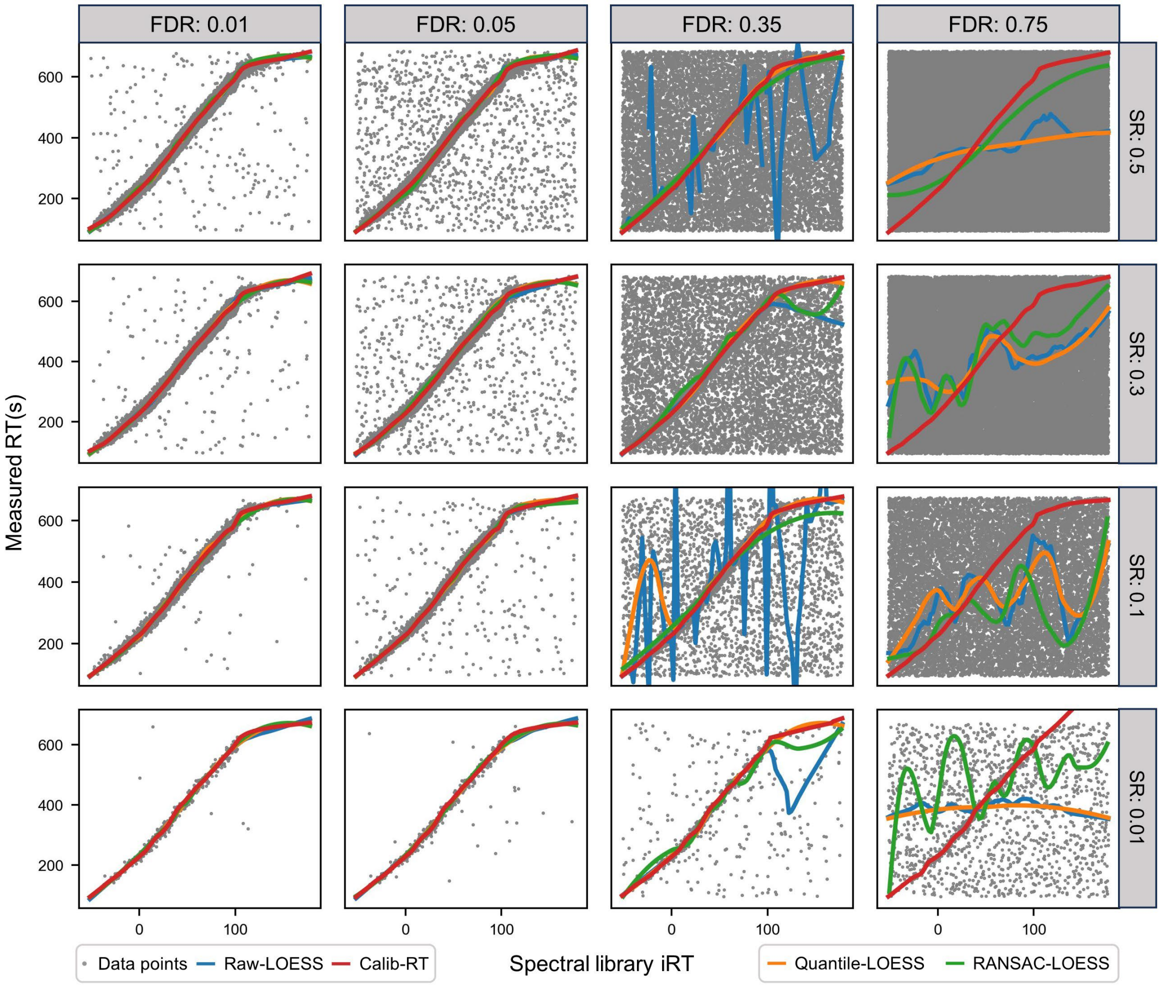

SR	FDR	#points	MRD				Time(s)			
			Raw-LOESS	Quantile-LOESS	RANSAC-LOESS	Calib-RT	Raw-LOESS	Quantile-LOESS	RANSAC-LOESS	Calib-RT
0.50	0.01	23,261	0.03	0.03	0.03	0.03	0.15	1.71	2.08	0.03
	0.05	24,240	0.03	0.03	0.03	0.03	0.14	2.15	5.59	0.03
	0.35	35,428	0.14	0.03	0.04	0.03	0.14	4.90	78.23	0.03
	0.75	92,112	0.27	0.28	0.11	0.03	0.15	1.53	77.36	0.04
0.30	0.01	13,957	0.03	0.03	0.03	0.03	0.13	1.65	2.06	0.03
	0.05	14,545	0.03	0.03	0.03	0.03	0.14	3.11	6.28	0.03
	0.35	21,257	0.03	0.03	0.06	0.03	0.14	5.39	78.67	0.03
	0.75	55,268	0.29	0.24	0.24	0.03	0.14	1.68	78.60	0.03
0.10	0.01	4,654	0.03	0.03	0.03	0.03	0.12	2.73	3.64	0.03
	0.05	4,850	0.03	0.03	0.03	0.03	0.12	2.01	2.17	0.03
	0.35	7,088	0.37	0.20	0.06	0.03	0.12	4.58	79.07	0.03
	0.75	18,428	0.26	0.29	0.21	0.03	0.13	5.08	78.75	0.03
0.01	0.01	467	0.03	0.03	0.03	0.03	0.10	2.27	2.30	0.03
	0.05	487	0.03	0.03	0.03	0.03	0.11	2.30	5.97	0.03
	0.35	711	0.04	0.03	0.06	0.03	0.10	4.17	77.91	0.03
	0.75	1,848	0.37	0.37	0.52	0.03	0.10	1.34	78.74	0.02

d) Distortion-End-II dataset

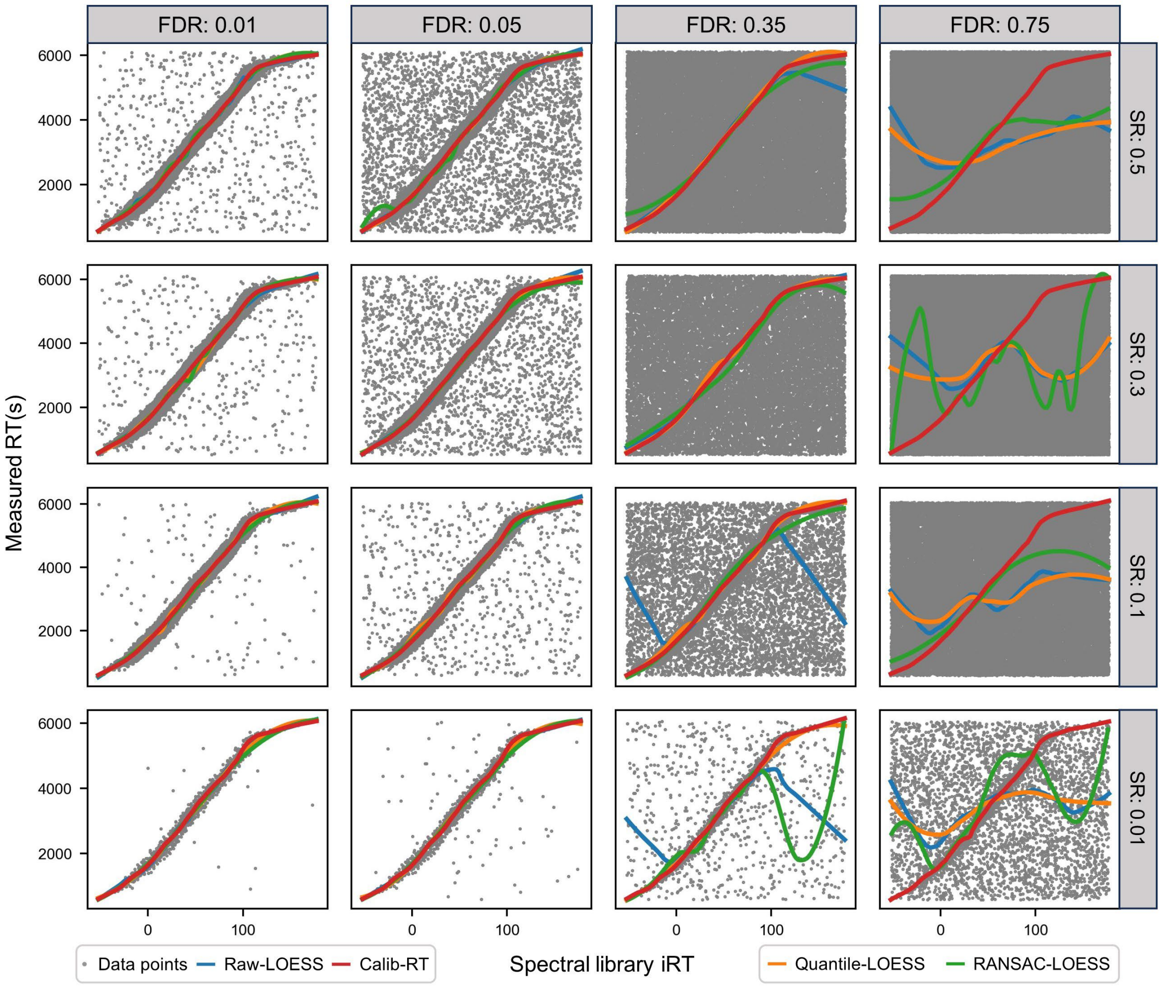

SR	FDR	#points	MRD				Time(s)			
			Raw-LOESS	Quantile-LOESS	RANSAC-LOESS	Calib-RT	Raw-LOESS	Quantile-LOESS	RANSAC-LOESS	Calib-RT
0.50	0.01	61,799	0.05	0.04	0.04	0.04	0.45	1.53	2.28	0.04
	0.05	64,402	0.04	0.04	0.05	0.04	0.44	2.93	7.37	0.04
	0.35	94,125	0.05	0.05	0.07	0.04	0.44	3.86	78.12	0.04
	0.75	244,724	0.33	0.37	0.16	0.04	0.43	1.57	78.01	0.06
0.30	0.01	37,080	0.04	0.05	0.05	0.04	0.37	3.34	3.65	0.03
	0.05	38,642	0.04	0.04	0.04	0.04	0.36	2.22	3.71	0.03
	0.35	56,476	0.05	0.05	0.08	0.04	0.35	4.60	78.24	0.04
	0.75	146,836	0.38	0.37	0.42	0.04	0.37	1.62	79.15	0.05
0.10	0.01	12,361	0.04	0.04	0.04	0.04	0.20	2.16	2.10	0.03
	0.05	12,882	0.04	0.05	0.04	0.04	0.21	3.12	5.14	0.03
	0.35	18,827	0.12	0.05	0.05	0.04	0.20	4.28	78.46	0.03
	0.75	48,948	0.29	0.32	0.10	0.04	0.21	1.37	78.56	0.03
0.01	0.01	1,238	0.04	0.04	0.04	0.04	0.11	2.26	3.61	0.03
	0.05	1,290	0.04	0.04	0.04	0.04	0.11	3.55	6.16	0.03
	0.35	1,885	0.15	0.04	0.12	0.04	0.11	4.26	78.48	0.03
	0.75	4,900	0.30	0.32	0.22	0.04	0.11	1.58	78.05	0.03

e) Exp-I dataset

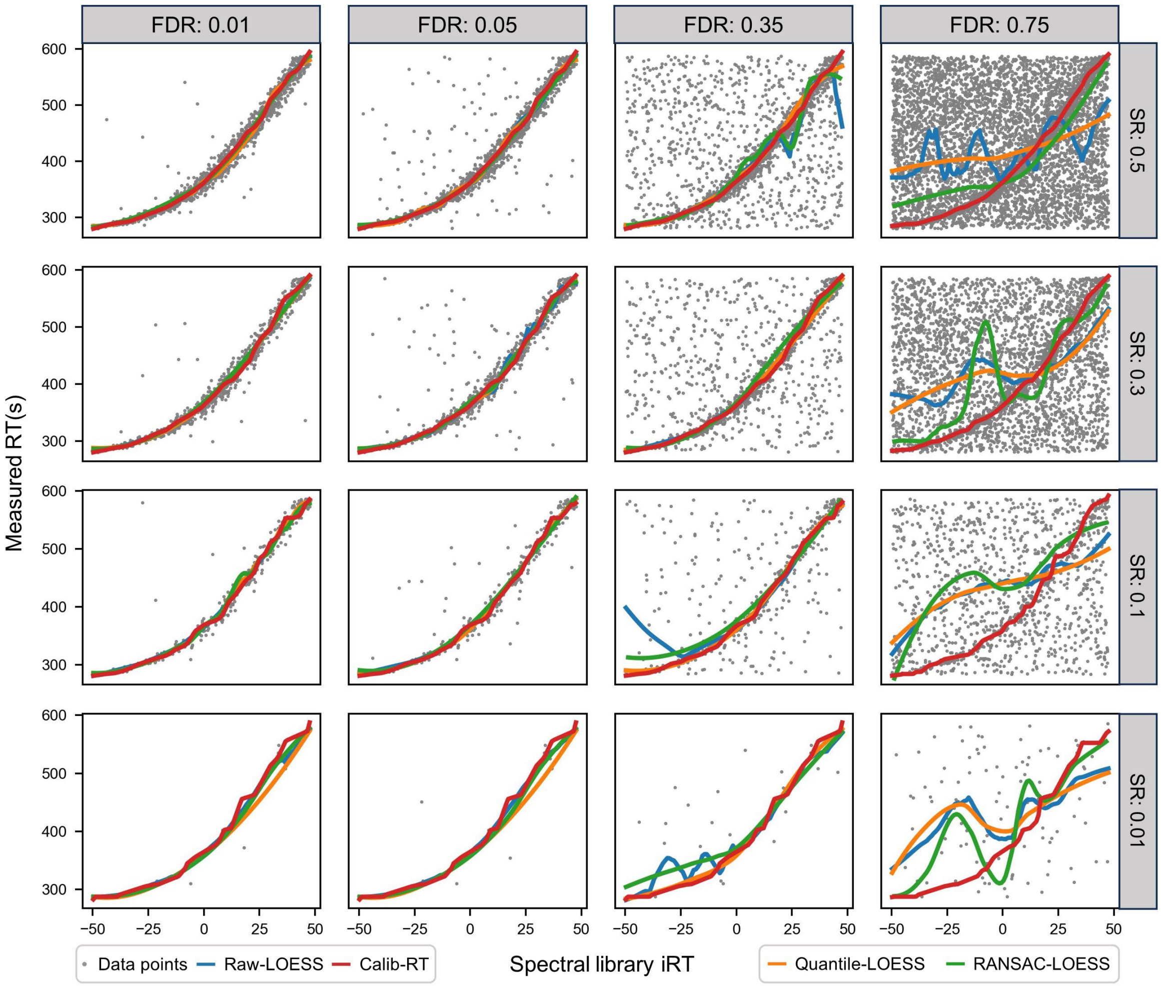

SR	FDR	#points	MRD				Time(s)			
			Raw-LOESS	Quantile-LOESS	RANSAC-LOESS	Calib-RT	Raw-LOESS	Quantile-LOESS	RANSAC-LOESS	Calib-RT
0.50	0.01	1,572	0.02	0.02	0.02	0.02	0.11	3.45	2.21	0.06
	0.05	1,638	0.02	0.02	0.02	0.02	0.10	4.23	6.73	0.06
	0.35	2,394	0.03	0.02	0.03	0.02	0.25	6.91	127.77	0.05
	0.75	6,224	0.15	0.15	0.06	0.02	0.25	2.24	126.58	0.05
0.30	0.01	944	0.02	0.02	0.02	0.02	0.24	5.68	6.07	0.06
	0.05	984	0.02	0.02	0.02	0.02	0.24	3.69	11.97	0.06
	0.35	1,437	0.02	0.02	0.02	0.02	0.24	9.03	103.72	0.03
	0.75	3,736	0.15	0.15	0.11	0.02	0.10	1.51	83.81	0.03
0.10	0.01	316	0.02	0.02	0.02	0.02	0.10	2.61	2.15	0.03
	0.05	329	0.02	0.02	0.02	0.02	0.10	2.33	6.11	0.03
	0.35	480	0.04	0.02	0.04	0.02	0.10	4.73	77.31	0.03
	0.75	1,248	0.16	0.17	0.16	0.02	0.10	1.38	78.64	0.02
0.01	0.01	34	0.02	0.03	0.02	0.02	0.04	1.34	1.47	0.00
	0.05	35	0.02	0.03	0.02	0.02	0.04	1.33	2.66	0.00
	0.35	51	0.03	0.02	0.06	0.02	0.05	1.88	4.31	0.01
	0.75	132	0.18	0.19	0.09	0.01	0.10	1.62	78.33	0.02

f) Exp-II dataset

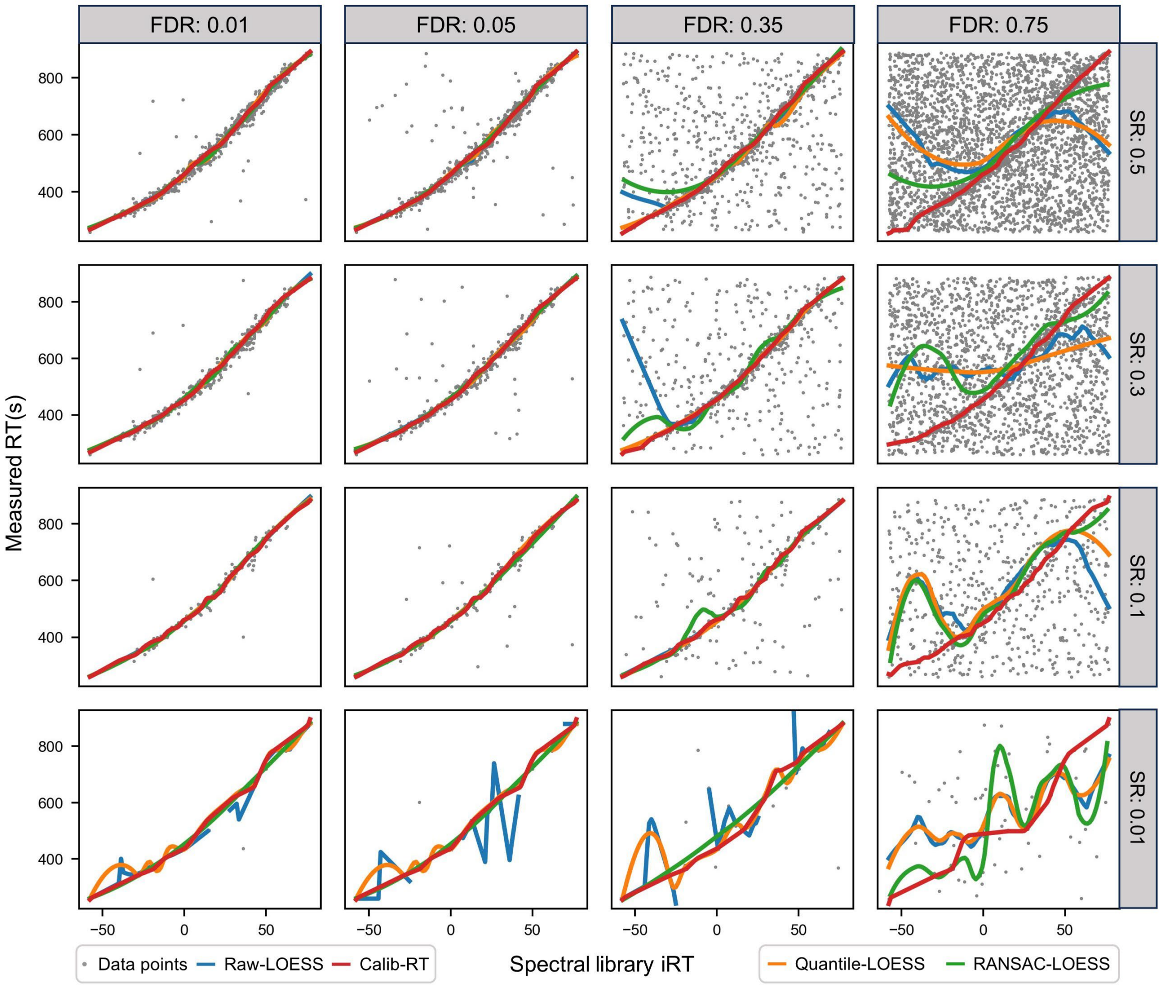

SR	FDR	#points	MRD				Time(s)			
			Raw-LOESS	Quantile-LOESS	RANSAC-LOESS	Calib-RT	Raw-LOESS	Quantile-LOESS	RANSAC-LOESS	Calib-RT
0.50	0.01	934	0.02	0.02	0.02	0.02	0.11	3.14	5.12	0.03
	0.05	973	0.02	0.02	0.02	0.02	0.11	2.48	9.94	0.03
	0.35	1,422	0.03	0.02	0.04	0.02	0.11	4.44	76.31	0.03
	0.75	3,696	0.15	0.17	0.08	0.02	0.10	1.37	78.46	0.03
0.30	0.01	561	0.02	0.02	0.02	0.02	0.10	2.65	3.01	0.03
	0.05	585	0.02	0.02	0.02	0.02	0.11	3.03	3.68	0.03
	0.35	854	0.05	0.02	0.05	0.02	0.10	4.33	77.60	0.03
	0.75	2,220	0.19	0.21	0.18	0.02	0.10	1.62	77.69	0.03
0.10	0.01	188	0.02	0.02	0.02	0.02	0.10	3.75	2.17	0.02
	0.05	196	0.02	0.02	0.02	0.02	0.10	2.64	5.82	0.02
	0.35	287	0.02	0.02	0.04	0.02	0.10	4.07	67.54	0.02
	0.75	744	0.16	0.14	0.11	0.02	0.10	5.74	77.89	0.02
0.01	0.01	21	0.02	0.01	0.02	0.02	0.02	0.83	1.00	0.00
	0.05	22	0.00	0.01	0.02	0.02	0.02	0.86	0.97	0.00
	0.35	31	0.01	0.02	0.04	0.03	0.04	1.10	3.24	0.00
	0.75	80	0.22	0.22	0.14	0.05	0.08	1.34	75.37	0.01

g) Linear-I dataset

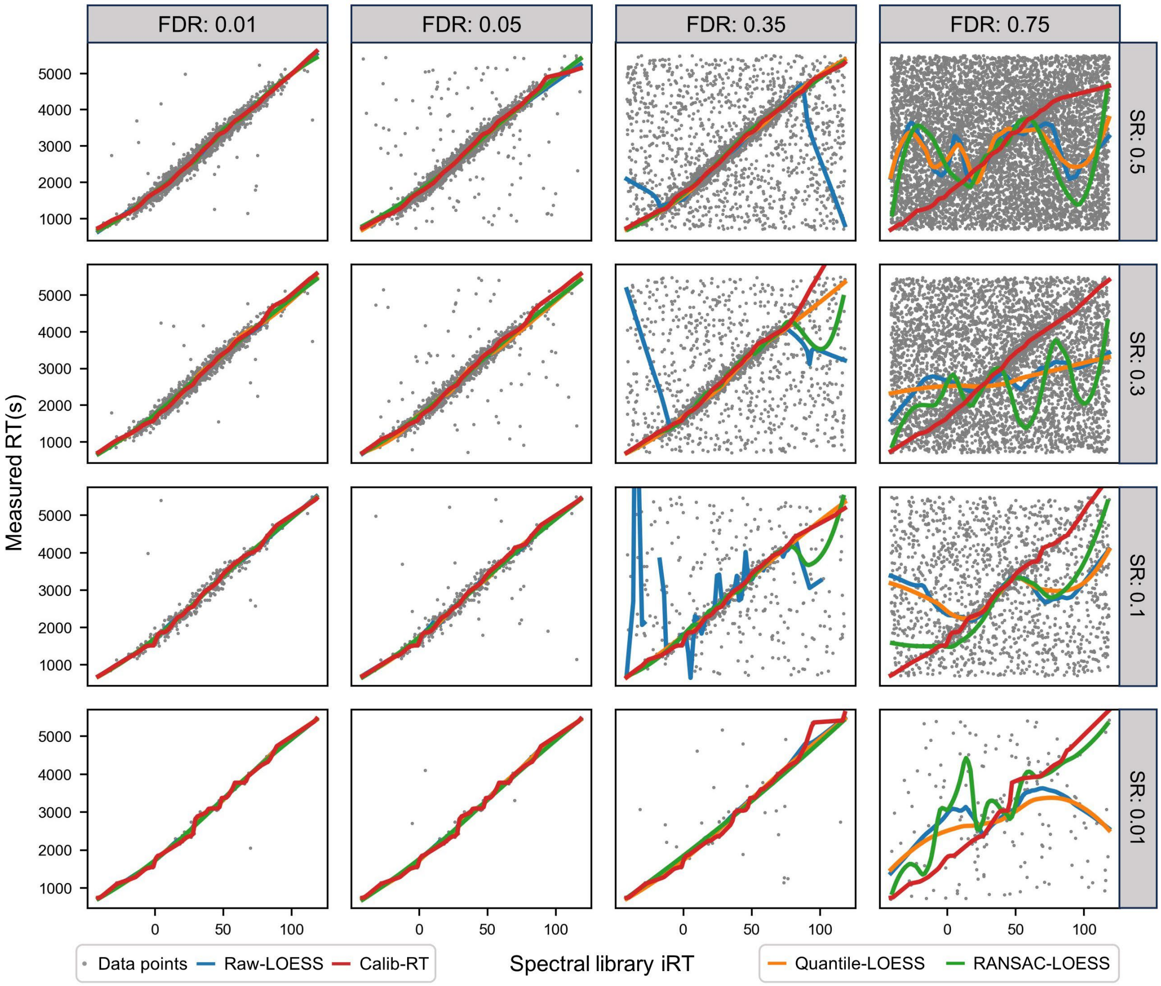

SR	FDR	#points	MRD				Time(s)			
			Raw-LOESS	Quantile-LOESS	RANSAC-LOESS	Calib-RT	Raw-LOESS	Quantile-LOESS	RANSAC-LOESS	Calib-RT
0.50	0.01	2,280	0.04	0.04	0.04	0.04	0.12	1.69	2.11	0.03
	0.05	2,376	0.04	0.04	0.04	0.04	0.12	2.32	11.91	0.03
	0.35	3,473	0.06	0.04	0.04	0.04	0.12	3.99	77.97	0.03
	0.75	9,028	0.24	0.26	0.21	0.04	0.11	1.61	78.64	0.02
0.30	0.01	1,369	0.04	0.04	0.04	0.04	0.11	2.46	2.10	0.03
	0.05	1,427	0.04	0.04	0.04	0.04	0.11	2.52	5.28	0.03
	0.35	2,085	0.10	0.04	0.05	0.04	0.11	4.81	79.34	0.03
	0.75	5,420	0.26	0.26	0.28	0.04	0.12	1.83	78.62	0.03
0.10	0.01	458	0.04	0.04	0.04	0.04	0.10	1.76	2.10	0.02
	0.05	477	0.04	0.04	0.04	0.04	0.10	4.44	7.58	0.02
	0.35	697	0.11	0.04	0.05	0.04	0.10	4.06	78.90	0.02
	0.75	1,812	0.22	0.21	0.13	0.04	0.10	1.40	77.99	0.03
0.01	0.01	48	0.03	0.03	0.03	0.02	0.05	1.23	2.91	0.01
	0.05	50	0.03	0.03	0.04	0.02	0.05	1.28	4.26	0.01
	0.35	73	0.04	0.03	0.05	0.03	0.07	2.99	73.57	0.01
	0.75	188	0.24	0.22	0.21	0.04	0.09	2.03	78.68	0.02

h) Linear-II dataset

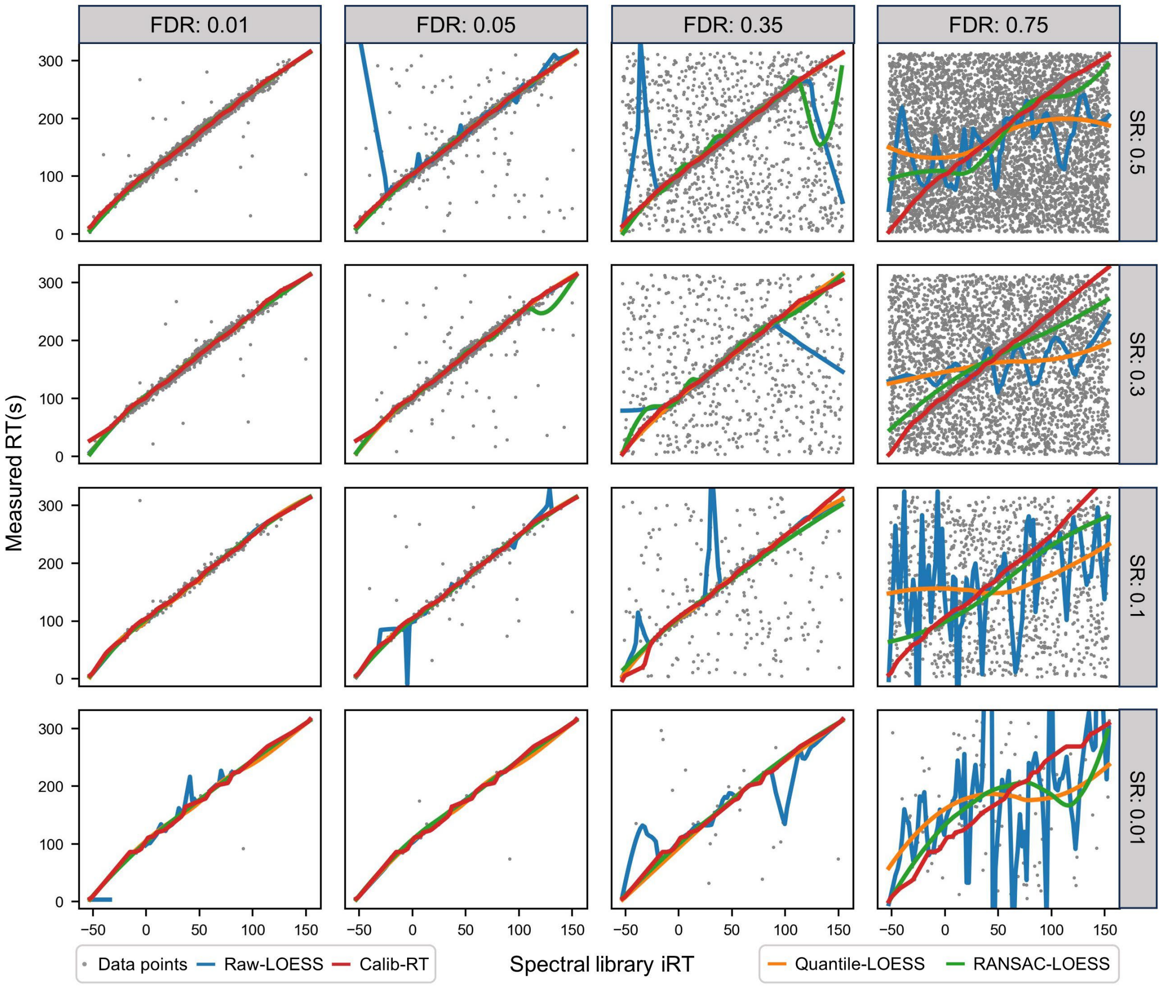

SR	FDR	#points	MRD				Time(s)			
			Raw-LOESS	Quantile-LOESS	RANSAC-LOESS	Calib-RT	Raw-LOESS	Quantile-LOESS	RANSAC-LOESS	Calib-RT
0.50	0.01	1,854	0.03	0.03	0.03	0.03	0.11	2.10	5.96	0.03
	0.05	1,932	0.24	0.03	0.03	0.03	0.11	2.27	5.94	0.03
	0.35	2,824	0.09	0.03	0.05	0.03	0.11	3.59	54.81	0.03
	0.75	7,340	0.23	0.23	0.17	0.03	0.11	1.36	78.10	0.03
0.30	0.01	1,113	0.03	0.03	0.03	0.04	0.10	2.15	5.27	0.02
	0.05	1,159	0.03	0.03	0.03	0.04	0.11	2.39	9.57	0.03
	0.35	1,694	0.10	0.03	0.04	0.03	0.10	4.01	78.37	0.03
	0.75	4,404	0.34	0.32	0.13	0.03	0.10	1.43	80.73	0.02
0.10	0.01	372	0.03	0.03	0.03	0.03	0.10	2.84	2.26	0.02
	0.05	388	0.03	0.03	0.03	0.03	0.10	2.60	6.20	0.02
	0.35	567	0.12	0.03	0.04	0.03	0.10	4.07	80.86	0.02
	0.75	1,472	0.41	0.39	0.14	0.03	0.10	1.35	84.42	0.02
0.01	0.01	39	0.01	0.03	0.03	0.03	0.04	1.63	3.17	0.00
	0.05	40	0.03	0.03	0.03	0.03	0.04	2.01	3.22	0.00
	0.35	59	0.08	0.04	0.05	0.03	0.06	2.50	47.36	0.01
	0.75	152	0.31	0.71	0.20	0.04	0.09	1.42	81.28	0.01

i) S-I dataset

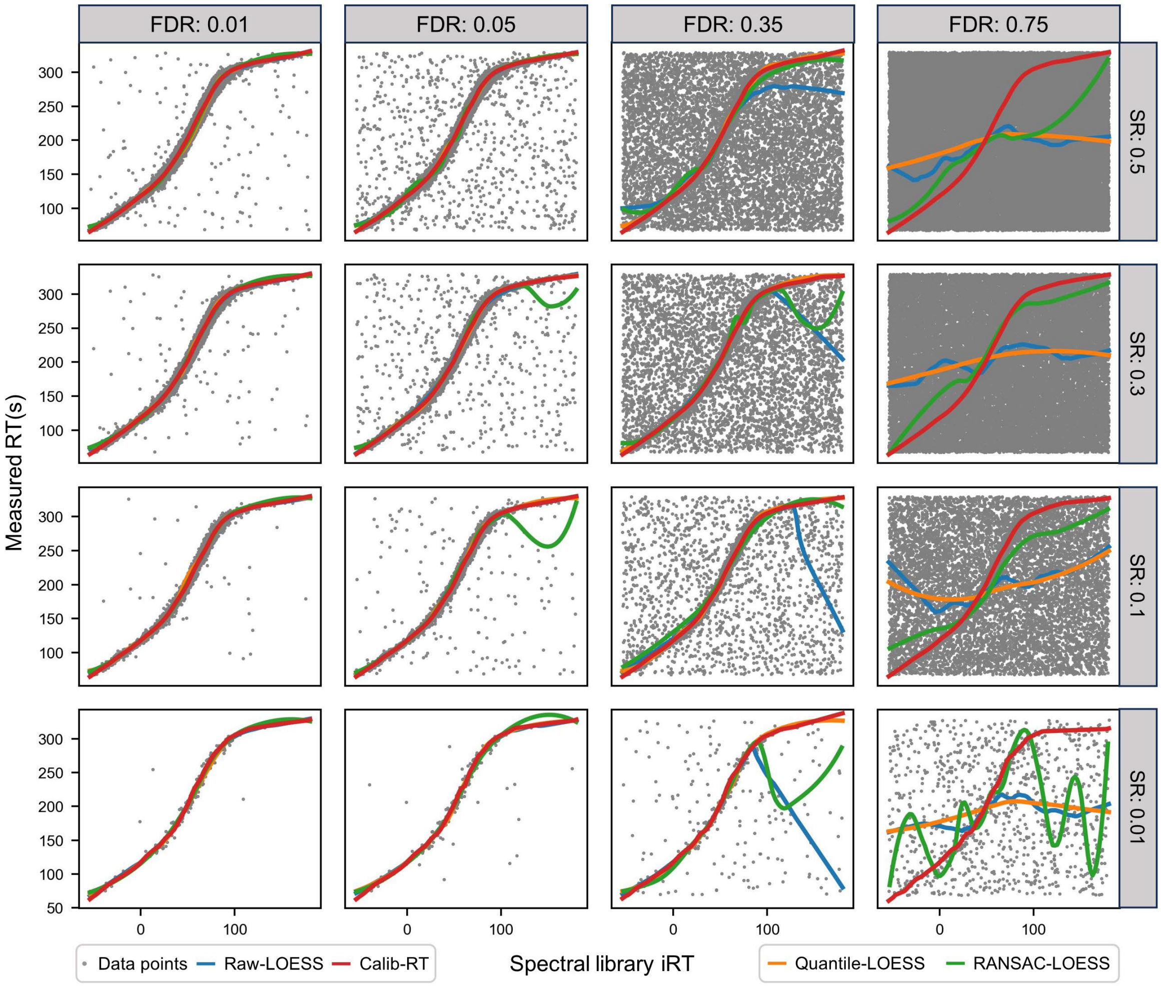

SR	FDR	#points	MRD				Time(s)			
			Raw-LOESS	Quantile-LOESS	RANSAC-LOESS	Calib-RT	Raw-LOESS	Quantile-LOESS	RANSAC-LOESS	Calib-RT
0.50	0.01	14,896	0.02	0.02	0.02	0.02	0.15	2.29	3.73	0.10
	0.05	15,524	0.02	0.02	0.03	0.02	0.11	2.94	2.34	0.04
	0.35	22,688	0.06	0.02	0.04	0.02	0.14	4.72	99.46	0.05
	0.75	58,988	0.24	0.31	0.17	0.02	0.28	2.32	128.90	0.05
0.30	0.01	8,939	0.02	0.02	0.02	0.02	0.27	3.54	5.91	0.05
	0.05	9,315	0.02	0.02	0.02	0.02	0.27	7.64	5.85	0.05
	0.35	13,614	0.03	0.02	0.03	0.02	0.27	5.66	44.34	0.05
	0.75	35,396	0.31	0.33	0.11	0.02	0.27	2.23	90.54	0.05
0.10	0.01	2,981	0.02	0.02	0.02	0.02	0.25	3.16	3.51	0.05
	0.05	3,107	0.02	0.02	0.03	0.02	0.26	5.92	8.41	0.05
	0.35	4,540	0.03	0.02	0.06	0.02	0.26	6.90	127.17	0.05
	0.75	11,804	0.30	0.33	0.12	0.02	0.26	2.31	128.04	0.05
0.01	0.01	299	0.02	0.02	0.02	0.02	0.23	4.53	6.13	0.04
	0.05	312	0.02	0.02	0.02	0.02	0.22	5.79	6.08	0.04
	0.35	456	0.05	0.02	0.05	0.02	0.23	8.31	128.74	0.04
	0.75	1,184	0.24	0.28	0.16	0.02	0.23	2.24	103.67	0.02

j) S-II dataset

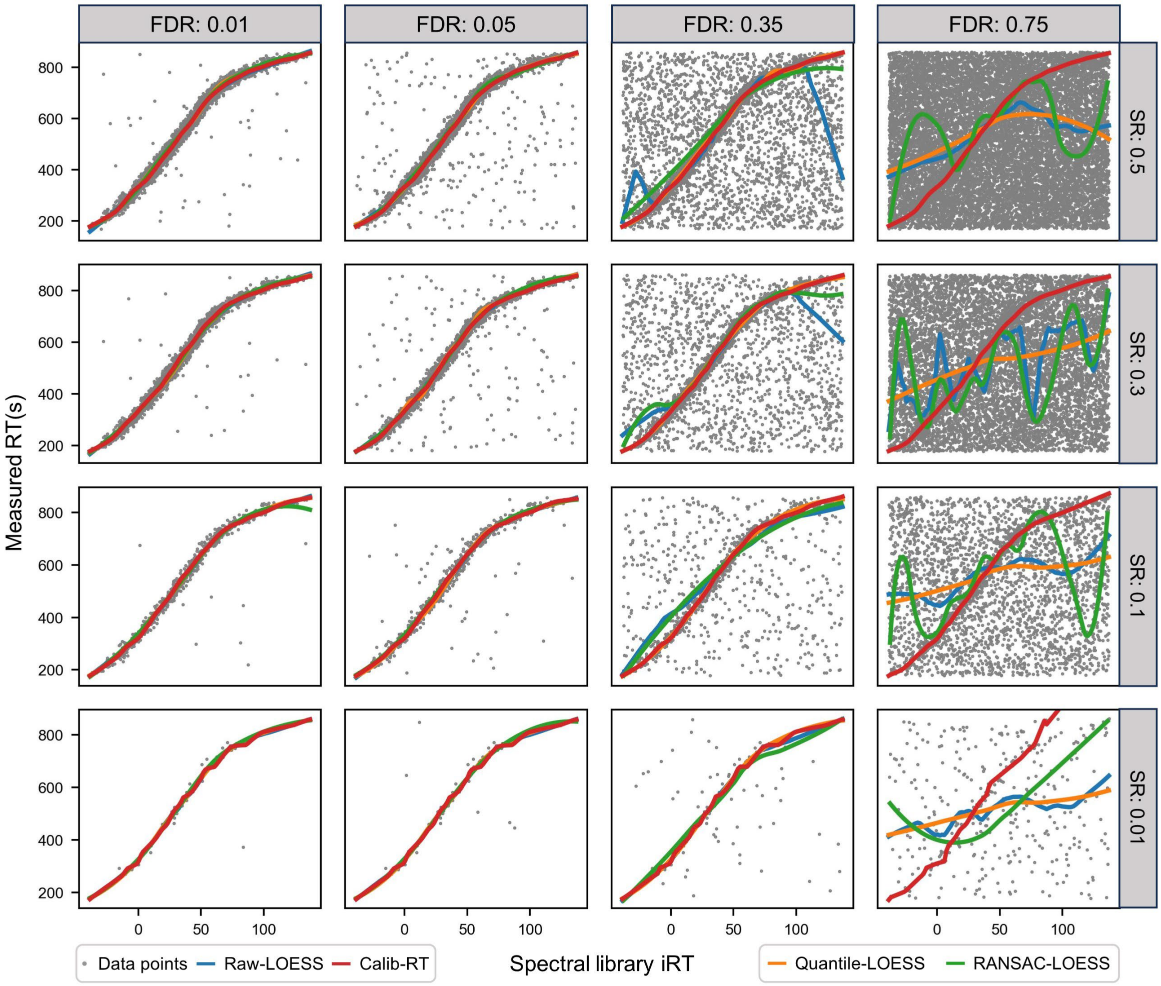

SR	FDR	#points	MRD				Time(s)			
			Raw-LOESS	Quantile-LOESS	RANSAC-LOESS	Calib-RT	Raw-LOESS	Quantile-LOESS	RANSAC-LOESS	Calib-RT
0.50	0.01	4,493	0.02	0.02	0.02	0.02	0.17	3.88	4.82	0.04
	0.05	4,683	0.03	0.02	0.03	0.02	0.28	3.57	5.50	0.03
	0.35	6,844	0.06	0.02	0.08	0.02	0.12	5.04	80.58	0.03
	0.75	17,792	0.20	0.23	0.27	0.02	0.12	1.69	78.10	0.04
0.30	0.01	2,696	0.03	0.03	0.02	0.02	0.12	2.64	2.18	0.04
	0.05	2,810	0.03	0.03	0.03	0.02	0.11	3.07	5.94	0.04
	0.35	4,107	0.06	0.02	0.07	0.02	0.11	4.22	77.63	0.04
	0.75	10,676	0.22	0.24	0.25	0.02	0.12	2.57	78.91	0.03
0.10	0.01	900	0.02	0.02	0.03	0.02	0.10	2.60	3.84	0.03
	0.05	938	0.02	0.02	0.02	0.02	0.11	2.42	2.25	0.03
	0.35	1,371	0.09	0.02	0.09	0.02	0.10	4.24	77.27	0.03
	0.75	3,564	0.24	0.26	0.15	0.02	0.11	2.81	78.75	0.03
0.01	0.01	91	0.02	0.02	0.02	0.02	0.09	2.68	3.69	0.01
	0.05	95	0.02	0.02	0.02	0.02	0.09	3.05	7.25	0.01
	0.35	139	0.02	0.02	0.05	0.02	0.09	4.42	67.78	0.02
	0.75	360	0.26	0.28	0.27	0.05	0.09	1.37	77.04	0.02

Fig. 2. The performance comparison between Calib-RT and the other three algorithms on test datasets. "SR" (sampling rate) refers to the ratio of randomly extracting data points from noise-free ground truth data points. "FDR" (false discovery rate) is the ratio of the number of noise points to the number of all data points. "MRD" means the mean relative deviation between measured RTs and predicted RTs which only considers the data points without noise. Each subplot (including 4*4 sub-subplots) accompanied with a table corresponds to a dataset. a) Distortion-Begin-I dataset. b) Distortion-Begin-II dataset. c) Distortion-End-I dataset. d) Distortion-End-II dataset. e) Exp-I dataset. f) Exp-II dataset. g) Linear-I dataset. h) Linear-II dataset. i) S-I dataset. j) S-II dataset.

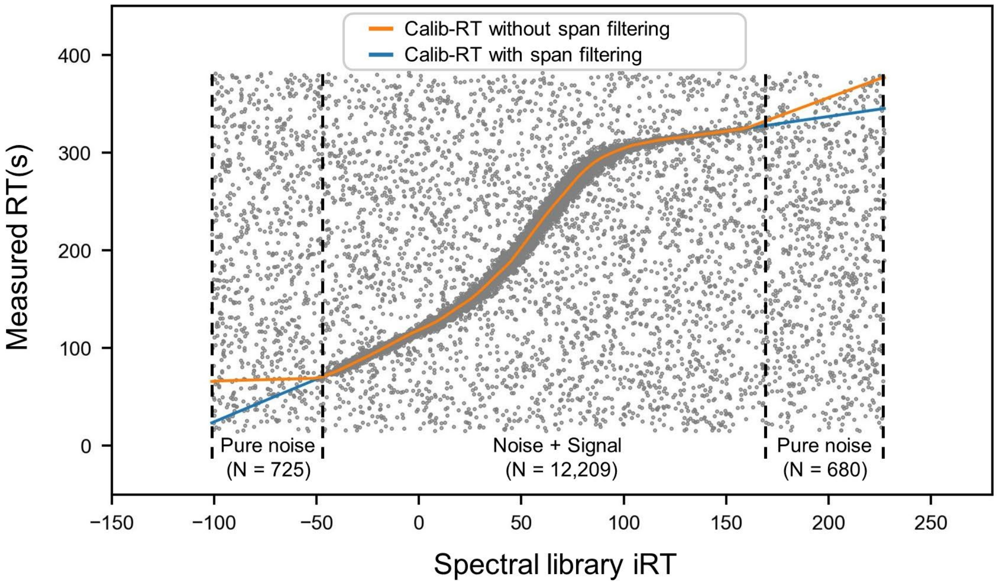

Fig. 3. The use of span filtering affects the performance of Calib-RT in the pure noise interval on S-I dataset (sampling rate: 0.3, noise level: 35% FDR). Calib-RT with span filtering tends to use data, while it without span filtering tends to use linear extrapolation to finish the fitting in the pure noise interval.
